# Blockage of glycolysis by targeting PFKFB3 suppresses tumor growth and metastasis in head and neck squamous cell carcinoma

**DOI:** 10.1186/s13046-016-0481-1

**Published:** 2017-01-07

**Authors:** Hui-Min Li, Jie-Gang Yang, Zhuo-Jue Liu, Wei-Ming Wang, Zi-Li Yu, Jian-Gang Ren, Gang Chen, Wei Zhang, Jun Jia

**Affiliations:** 1 The State Key Laboratory Breeding Base of Basic Science of Stomatology (Hubei-MOST) and Key Laboratory of Oral Biomedicine Ministry of Education, School and Hospital of Stomatology, Wuhan University, Wuhan, 430079 China; 2 Department of Oral and Maxillofacial Surgery, School and Hospital of Stomatology, Wuhan University, No237, Luoyu Road, Hongshan District, Wuhan, 430079 China; 3 Oral Medical Center, Xiangya Hospital, Central South University, Changsha, Hunan 410000 China

**Keywords:** HNSCC, Glycolysis, PFKFB3, Metastasis, Invadopodia, Invasion

## Abstract

**Background:**

Many cancers including head and neck squamous cell carcinoma (HNSCC) are characterized by a metabolic rewiring with increased glucose uptake and lactate production, termed as aerobic glycolysis. Targeting aerobic glycolysis presents a promising strategy for cancer therapy. This study investigates the therapeutic potential of glycolysis blockage by targeting phosphofructokinase-2/fructose-2, 6-bisphosphatase 3 (PFKFB3) in HNSCC.

**Methods:**

1-(4-pyridinyl)-3-(2-quinolinyl)-2-propen-1-one (PFK15) was used as a selective antagonist of PFKFB3. Glycolytic flux was determined by measuring glucose uptake, lactate production and ATP yield. PFKFB3 expression was examined using HNSCC tissue arrays. Cell proliferation, apoptosis and motility were analysed. HNSCC xenograft mouse model and metastasis mouse model were established to examine the therapeutic efficacy of PFK15 in vivo.

**Results:**

HNSCC showed an increased PFKFB3 expression compared with adjacent mucosal tissues (*P* < 0.01). Targeting PFKFB3 *via* PFK15 significantly reduced the glucose uptake, lactate production and ATP generation in HNSCC cell lines. PFK15 suppressed cell proliferation, halted cell cycle progression and induced cell apoptosis. The invadopodia of HNSCC cells was markedly reduced after PFK15 treatment, thereby impairing cell motility and extracellular matrix degradation ability. The in vivo data from the xenograft mice models proved that PFK15 administration suppressed the tumor growth. And the results from the metastatic mice models showed administration of PFK15 alleviated the lung metastasis of HNSCC and extended the life expectancy of mice.

**Conclusions:**

The pharmacological inhibition of PFKFB3 *via* PFK15 suppressed tumor growth and alleviated metastasis in HNSCC, offering a promising strategy for cancer therapy.

**Electronic supplementary material:**

The online version of this article (doi:10.1186/s13046-016-0481-1) contains supplementary material, which is available to authorized users.

## Background

Aberrant glucose metabolism is the most common biochemical characteristic of cancer cells [1]. Compared with normal (quiescent) cells, cancer cells exhibit aerobic glycolysis or the ‘Warburg effect’, which is characterised by an increased glucose uptake and lactate production through a glycolytic pathway even in the presence of sufficient oxygen [2]. Although the efficacy of ATP generation by glycolysis is low, the intermediates produced by glycolysis which shunt into biosynthetic pathways support the rapid proliferation and survival of cancer cells, thereby promoting tumor occurrence and progression. The conversion of fructose-6-phospate to fructose 1, 6-bisphosphate is the primary rate-limiting and irreversible reaction amongst a series of reactions in glycolysis; this reaction is catalysed by phosphofructokinase-1 (PFK1), which serves as the prominent pacemaker during the process. Fructose 2, 6-bisphosphate (F26BP) can override the inhibition by ATP and increased glucose uptake by interacting with PFK1. Phosphofructokinase-2/fructose-2, 6-bisphosphatase 3 (PFKFB3) bears an oncogene-like regulatory element and benefits the synthesis of F26BP to promote glycolytic flux with its high kinase activity [3, 4]. Given that this gene is commonly overexpressed in human cancers, including breast, colon, ovarian and thyroid carcinomas [5], but is insufficiently expressed in normal tissues, targeting PFKFB3 presents a promising strategy for cancer treatment. The overexpression of PFKFB3 is fundamental to the targeted therapy of various cancer types [4]. However, whether PFKFB3 is overexpressed in head and neck squamous cell carcinoma (HNSCC) tissues remains unknown.

As the sixth most common malignancy of the leading neoplasms around the world [6], HNSCC affects six million new patients each year and accounts for over 90% of head and neck cancers [7, 8]. Most clinical HNSCC patients suffer from long-term poor prognoses and conditions that have not been improved substantially over the last three decades partially because of high local recurrence and frequent distant metastasis [7, 9]. Neoplastic invasion and metastasis require a strong extracellular matrix (ECM) degradation ability [10] that largely depends on the formation of a specialised subcellular structure, invadopodia in cancer cells [11]. De Bock et al. found that PFKFB3 affected directional migration and controlled the formation of lamellipodia/filopodia in endothelial cells by mechanistically compartmentalising with F-actin in these motile protrusions [12]. In addition, many studies revealed the significance of these F-actin-rich membrane protrusions during tumor metastasis [13, 14]. And a recent study indicated that glycolysis in tumor cells contributed to the assembly and stability of invadopodia in tumor cells [15]. Therefore, PFKFB3 may also control the protrusions in cancer cells, and inhibiting this gene presents a promising strategy for alleviating the aggressive behaviour of cancer cells.

PFKFB3 suppression can induce the apoptosis of cancer cells and halt cell cycles [16]. Moreover, the selective antagonist of PFKFB3 slows down the growth of transplanted tumor in several animal models [16–18]. However, the therapeutic effects of PFKFB3 suppression targeting cancer metastasis remain unknown. In this study, we investigated the effects of targeting PFKFB3 on the growth, apoptosis, migration and invasion of HNSCC cells, and further explored the possible roles of this gene in the assembly of functional invadopodia in HNSCC cell lines. We also tested the therapeutic efficacy of PFK15 by establishing HNSCC xenograft and metastasis nude mice models, and found that targeting PFKFB3 offered a promising therapeutic strategy not only for suppressing primary tumor growth but also for alleviating distant metastasis in HNSCC.

## Methods

### Chemicals and antibodies

Dulbecco’s modified Eagle’s medium (DMEM), DMEM/F12, fetal bovine serum (FBS), penicillin and streptomycin were obtained from GIBCO (Carlsbad, CA). 1-(4-pyridinyl)-3-(2-quinolinyl)-2-propen-1-one (PFK15; Selleck, Houston, TX), dimethylsulfoxide (DMSO; Sigma-Aldrich, St Louis, MO), Gelatin, propidium iodide (PI) and Ribonuclease A (Sigma-Aldrich, St Louis, MO), Mounting Medium with DAPI (Zhongshan, Beijing, China) were purchased. Matrigel^TM^ matrix (BD Biosciences, San Jose, CA), transwell Boyden chamber system and 6-well Ultralow Adherence plates (Corning life Sciences, Wilkes Barre, PA), 0.5% Triton X-100 (MP Biomedical, Solon, OH) were also used. Epithelial growth factor (EGF) and basic fibroblast growth factor (bFGF) were purchased from Peprotech (Rocky Hill, NJ). Antibodies including PFKFB3, phosphor-Rb, cyclinD1, cleaved-caspase3 (Cell Signaling, Danvers, MA), Bcl2 (Abcam, Cambridge, UK) and β-Actin (Santa Cruz, CA) were purchased. All other chemicals were classified as analytical grade reagents.

### Cell culture

HNSCC cell lines Cal27 and FaDu were purchased from the China Center for Type Culture Collection (CCTCC, Wuhan, China) and cultured according to the manufacturer’s instructions with high glucose DMEM containing 10% FBS, 100 U/ml penicillin, and 100 μg/ml streptomycin in a humidified atmosphere of 95% air and 5% CO_2_ at 37 °C. Cell viability was measured by the Vi-CELL cell viability analyzer (Beckman Coulter, Fullerton, CA).

### Human HNSCC tissues array and immunohistochemistry

One hundred eleven pathologically confirmed HNSCC specimens (including 33 lymph node metastatic samples) and 57 precancerous normal tissues were collected at the Hospital of Stomatology, Wuhan University. Then the fixed tissues were made into a HNSCC tissue arrays with the assistance of Kindstar Gobal Co. Ltd (Wuhan, China). The immunohistochemical experiment was performed according to our previous procedures [19]. All the procedures were performed in accordance with the guidelines of National Institutes of Health regarding the use of human tissues. Briefly, the sections were dewaxed in xylene, rehydrated in a graded series of ethanol and double-distilled water, and antigen retrieved by microwave. After incubation with 3% hydrogen superoxide and 10% normal goat serum for 15 min, the sections were then incubated overnight at 4 °C with monoclonal rabbit anti-human PFKFB3 (Abcam, Cambridge, UK, 1:1000). The antibody binding was detected by horseradish peroxidase-conjugated secondary antibody with a diaminobenzene substrate kit (Dako, Carpinteria, CA) according to the manufacturer’s protocol. The negative control slides were obtained by using PBS instead of the primary antibody. All slices were scanned by an Aperio ScanScope CS scanner (Epistem, Cambridge, MA) and quantified using Aperio Quantification software (Version 9.1, Epistem) for staining quantification as we previously reported [20]. Four random areas were selected for scanning and quantification. Histoscore of membrane and nuclear staining was calculated as a percentage of different positive cells using the formula (3+) × 3 + (2+) × 2 + (1+) × 1. Histoscore of pixel quantification was calculated as total intensity/total cell number. The threshold for scanning of different positive cells was set according to the standard controls provided by Aperio.

### Measurement of F26BP, glucose uptake, L-Lactate and ATP

The intracellular level of F26BP, glucose uptake, L-lactate and ATP output were detected according to manufacturers’ instructions. Details were shown in Additional file 1: Methods.

### Determination of viability, tumor spheres formation, cell cycle progression, apoptosis and apoptosis ability

Detailed procedures were shown in Additional file 1: Methods.

### Cell migration and invasion detection

Cell migration and invasion ability was detected by wound healing assay and transwell chamber assay, shown in Additional file 1: Methods.

### EdU (5’-ethynl-2’-deoxyuridine) staining assay

The effects of PFK15 on cell proliferation were assessed by the Cell-Light™ EdU Apollo®488 In Vitro Imaging Kit according to the manufacturer’s instructions. The number and proportion of the cells incorporated EdU was visualized and the fluorescence intensity was quantified using Image J1.42.

### Western blotting

Western blotting analysis was performed according to our previous procedures [21]. Briefly, Cal27 cells treated with indicated concentrations of PFK15 for 24 h were collected, precipitated and lysed. The concentration of protein was detected by BCA assays. 20 μg of protein with loading buffer were loaded on 10% SDS-PAGE gels and transferred PVDF membranes. The membranes were blocked with 5% nonfat dry milk for 1 h at room temperature and then probed with primary antibody overnight at 4 °C at dilutions recommended by the suppliers, including pRb (1:1000), cyclinD1 (1:500), cleaved-capase-3 (1:1000), Bcl2 (1:2000) and β-Actin (1:20000). The membranes were incubated with secondary antibody conjugated to horseradish peroxidase (HRP) for 1 h at room temperature. Then the membranes were incubated using Pico West chemiluminescent reagent.

### Invadopodia analysis and cell immunofluorescence staining

0.1% Gelatin was labeled by the fluorescent dye Alex 568 using Protein Labeling Kit according to the prescribed protocol. The cells after different treatment were fixed with 4% formaldehyde and permeabilized with 0.5% Triton X-100 in PBS. The cells were then blocked with 5% BSA for 1 h at room temperature and incubated with appropriate primary antibodies, PFKFB3 (1:50) and cortactin (1:100) overnight at 4 °C, followed by secondary antibodies conjugated with Dylight 649 and 408 for 1 h. The nuclei were stained with mounting medium with DAPI. After immunofluorescence staining, overhead and section images were captured by an objective confocal laser-scanning microscope. The number and area of invadopodia formed by the cells was determined and quantified using ImageJ1.42.

### Establishment and PFK15 treatment of Cal27 xenograft model

All animal studies of nude mice were approved and supervised by Animal Care and Use Committee of Wuhan University. Female athymic nude mice (18–20 g; 6-8weeks of age) were obtained from the Hunan SJA Laboratory Animal Co. Ltd (Changsha, Hunan, China). All the animal experiments have approved from the review board of the ethics committee of Hospital of Stomatology, Wuhan University, and supervised by the Animal Care and Use Committee of Wuhan University. For xenografts, approximately 1 × 10^7^ viable Cal27 cells were resuspended in 100 μl PBS solution and subcutaneously injected into 18 mice. After 2 weeks, the mice were divided into three groups randomly, which received intraperitoneal injection of PFK15 (10 mg/kg; *n* = 6), PFK15 (20 mg/kg; *n* = 6) or normal saline (vehicle, 100 μl; *n* = 6) three times per week for 2 weeks. The mice were monitored every other day for tumor volume by caliper measurements (Length × Width^2^)/2. The mice were euthanized and sacrificed at the indicated time points and the tumors were harvested for successive histology and molecular analysis according to standard procedures.

For metastatic models, 10 mice were injected with Cal27 cells (2 × 10^6^ cells resuspended in 100 μl PBS) *via* the tail vein. Two weeks after injection, mice were randomly divided into two groups and received intraperitoneal injection of normal saline (vehicle, 100 μl; *n* = 5), PFK15 (10 mg/kg; *n* = 5) three times per week for 2 weeks, then left for a long period for determining the survival rate. After 50 days of the first PFK administration, the mice were euthanized and the lung tumor nodules were counted after execution. Microscopic analysis of metastases was performed on the sections of formalin-fixed, paraffin-embedded lung tissues stained with hematoxylin and eosin (H & E) and pan-CK.

### Statistical analysis

All experiments were performed at least in triplicate and each experiment was repeated for three times. Data are presented as mean ± standard error of mean (SEM). Graphpad Prism Software (Graphpad Software, Inc) was used for accomplishing graphs. For statistical analysis between different treatment groups, a non-parametric two-sided *t*-test, and one-way ANOVA were used. Also a two-way ANOVA was used for group analysis. The differences were considered statistically significant if the *P*-value < 0.05.

## Results

### Abundant expression of PFKFB3 in HNSCC tissues

**Fig. 1:**
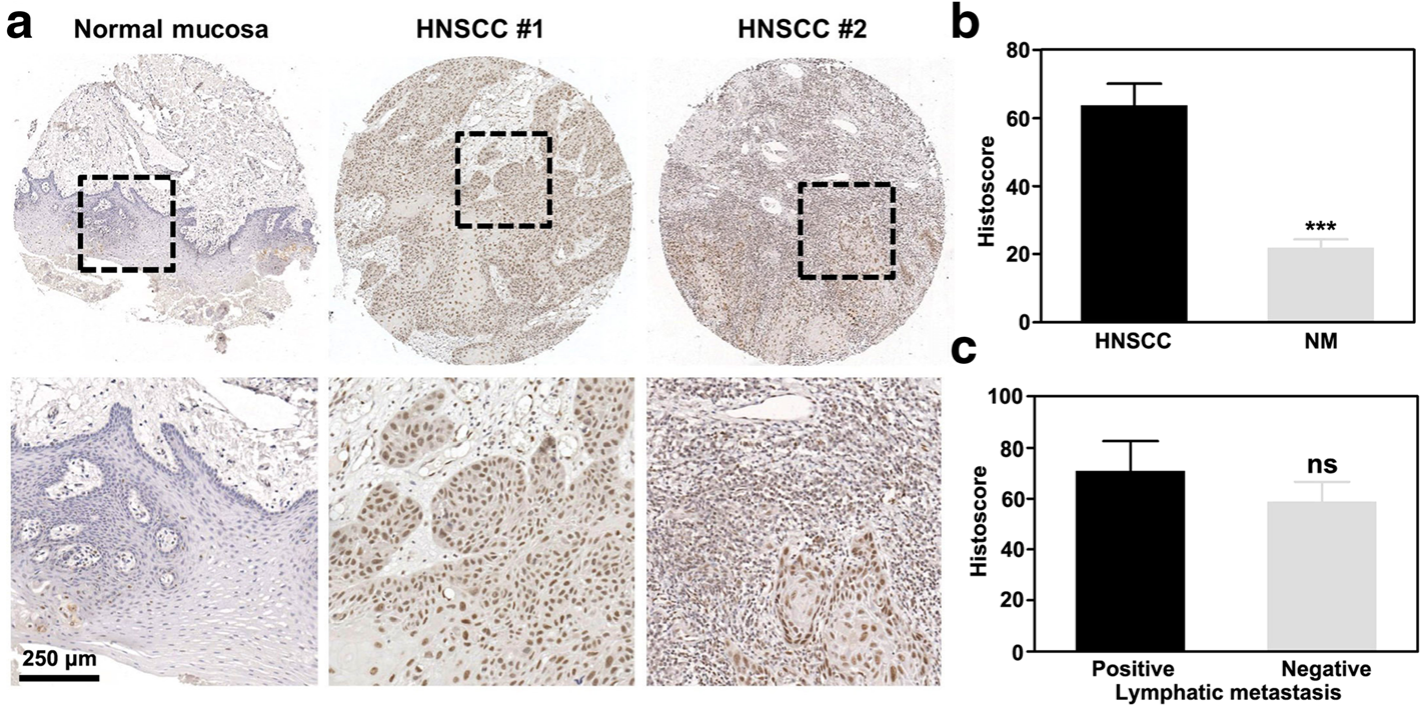
Abundant expression of PFKFB3 in human HNSCC. **a** Abundant expression of PFKFB3 in human HNSCC as compared with normal mucosa. **b** Histoscore of PFKFB3 in human HNSCC and normal mucosa. **c** Histoscore of PFKFB3 in the lymph node metastatic and non-metastatic HNSCC samples. Mean ± S.E.M.; ***P < 0.001

Therefore, we explored the expression of PFKFB3 in HNSCC using tissue arrays (*n* = 111), in which the normal adjacent tissues were used as control (*n* = 57, denoted as normal mucosa). As shown in Fig. 1, a dramatically enhanced immunohistochemical staining was found in most HNSCC tissues, and staining was almost negative in the normal mucosa tissues except in some basal layer cells. Most of the PFKFB3 positive staining was observed in the nuclei of the carcinoma cells, and cytoplasmic positive signals were detected in a much weaker level. Some stoma cells in cancer tissues were also PFKFB3 positive. By analysing the histoscores of PFKFB3 in both tumor and normal tissues, we observed a significantly higher expression of PFKFB3 in HNSCC tissues (Fig. 1b). We also analysed the PFKFB3 histoscores of the lymph node metastatic and non-metastatic samples (Fig. 1c). Although those tumors with positive metastasis had higher PFKFB3 histoscores than those with negative metastasis, no significant difference was observed between these samples. Therefore, PFKFB3 contributes to the development of HNSCC and may have a role in metastasis.

### PFK15 suppresses the glycolytic activity of HNSCC cells

**Fig. 2:**
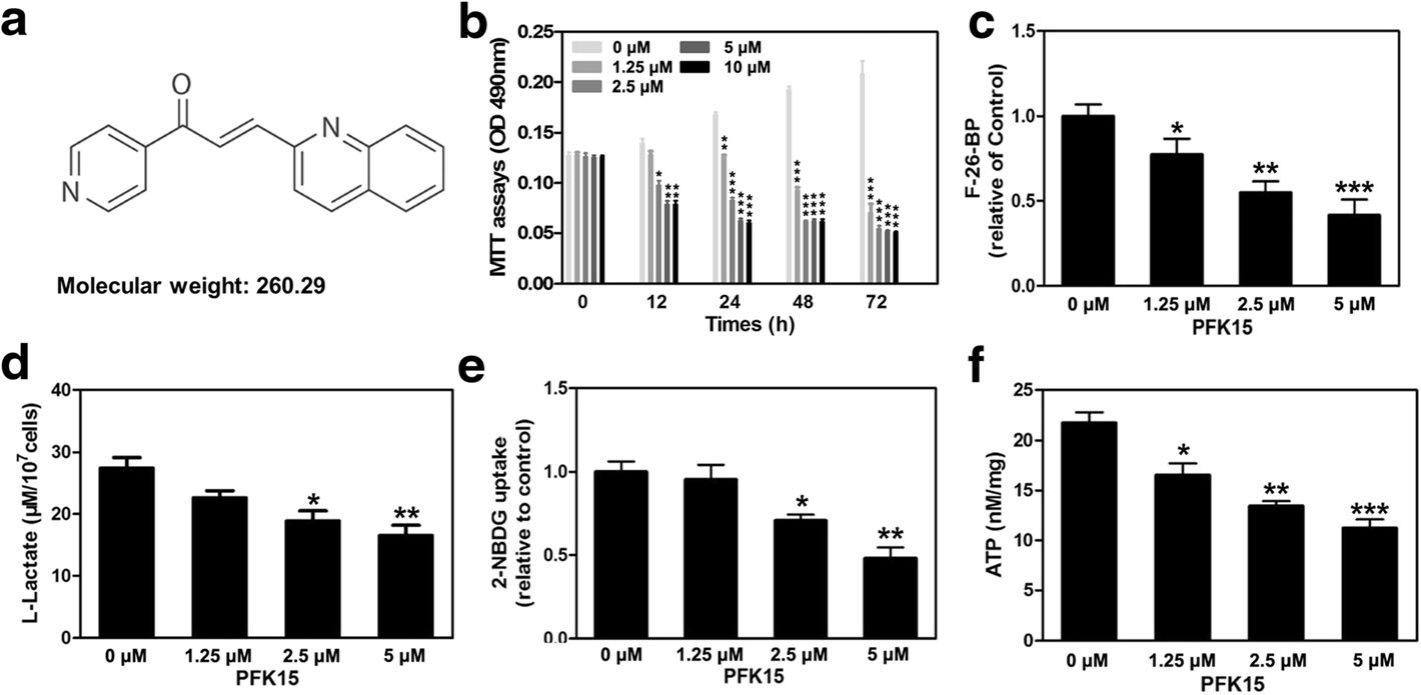
PFK15 suppresses the glycolytic activity of HNSCC cells. **a** Molecular structure of 1-(4-pyridinyl)-3-(2-quinolinyl)-2-propen-1-one (PFK15). **b** Cell growth determined by MTT assays in Cal27 cells after PFK15 treatment. **c** Measurement of fructose 2,6-bisphosphate (F26BP) in Cal27 cells after PFK15 treatment. **d** Glucose uptake was analyzed in Cal27 cells using 2-NBDG. **e** Lactate productionof Cal27 cells treated with PFK15 were detected. **f** ATP generation inside of the Cal27 cells treated with PFK15 were measured. Mean ± S.E.M.; *P < 0.05, **P < 0.01, ***P < 0.001

As a potent inhibitor of PFKFB3, PFK15 demonstrates approximately 100-fold more activities against PFKFB3 than 3-3 (3-pyridinyl)-1-(4-pyridinyl)-2-propen-1-one (3PO) (Fig. 2a) [16]. Using MTT assays, we analysed the effects of PFK15 on the growth of HNSCC cells, including Cal27 and FaDu cell lines. As shown in Fig. 2b, PFK15 treatment inhibited the cell growth of Cal27 in a concentration- and time-dependent manner. Similar results were obtained from the assays on FaDu cells (Additional file 1: Figure S1a). Given the critical role of PFKFB3 in glycolysis in most cancer cells by moderating the intercellular level of F26BP, we then verified the steady-state concentration of F26BP in HNSCC cells. As shown in Fig. 2c, the intracellular concentration of F26BP was dependently reduced in Cal27 cells. The intracellular lactate production, glucose uptake and intracellular ATP generation were then examined to measure the glycolytic activity of Cal27 cells after PFK15 treatment. As shown in Fig. 1d-e, the intracellular lactate production, glucose uptake and intracellular ATP generation in Cal27 cells were gradually decreased after PFK15 treatment. PFK15 also showed potent suppressive effects on the glycolytic activity of FaDu cells, represented as decreased lactate production, glucose uptake and ATP generation (Additional file 1: Figure S1b–d). Taken together, our data showed that PFK15 dramatically suppressed the glycolytic activity of HNSCC cells by impeding the production of F26BP.

### PFK15 suppresses cell proliferation, halts cell cycle and induces apoptosis in HNSCC cells

**Fig. 3:**
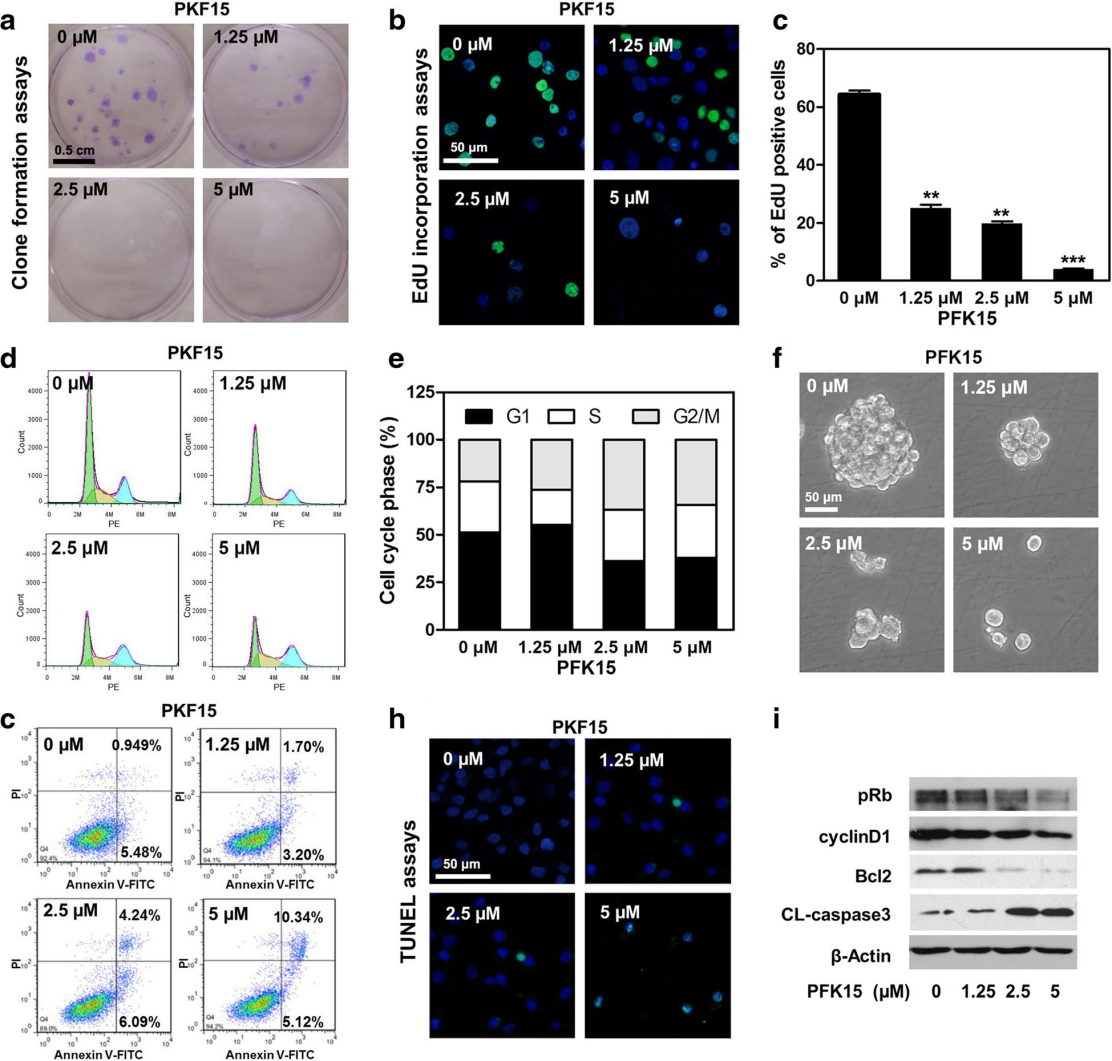
PFK15 suppresses cell proliferation, halts cell cycle and induces cell apoptosis in HNSCC cells. **a** PFK15 suppressed the colony formation of Cal27 cells in 2 weeks. **b** EdU incorporation assays indicated PFK15 inhibited the cell proliferation of Cal27 cells. **c** The quantitative data of the EdU incorporation assays. **d** PI staining revealed that PFK15 halted cell cycle progression and induced G2 phase arrest. **e** The quantitative data of cell cycle analysis based on Dean-Jett-Fox model. **f** Tumor sphere formation assays indicated PFK15 decreased the cancer stem cell population in Cal27 cells. **g** Annexin V-FITC/PI double staining demonstrated PFK15 induced moderate cell apoptosis of Cal27 cells. **h** TUNEL assays indicated PFK15 induced apoptotic cell death of Cal27 cells. **i** Protein expressions of phosphor-Rb (pRb), cyclin D1, Bcl2 and cleaved caspase3 (CL-caspase3) in PFK15 treated Cal27 cells were measured by western blots. Mean ± S.E.M.; **P < 0.01, ***P < 0.001

A high rate of glycolysis is required to support neoplastic growth not only by generating ATP but also by providing glycolytic intermediates for the synthesis of the necessary macromolecules for cancer cell mass duplication during division and proliferation [22]. Therefore, we examined the potential role of PFK15 in cell proliferation using colony formation and EdU incorporation assays. As shown in Fig. 3a, PFK15 significantly impaired the clone formation capability of Cal27 cells even at a relatively low concentration. At 2.5 μM and 5 μM concentrations, PFK15 almost entirely inhibited the clone formation of Cal27 cells. The EdU incorporation assays revealed the potent suppressive ability of PFK15 on cell proliferation presented as decreased EdU positive stained cells (Fig. 3b and c). The quantitative data suggested that even at 1.25 μM, PFK15 already suppressed more than 50% proliferative potentials of Cal27 cells. Similar results were obtained using FaDu cells (Additional file 1: Figure S2a and b). It was reported PFKFB3 could influence cell cycle transitions and determine cell fate in several previous studies [17, 23, 24]. We here also explored the effects of PFKFB3 suppression on the cell cycle progression and tumor spheres formation, which was usually used to determine the stemness of cancer cells. As shown in Fig. 3d and e, the cell population in the G1 phase was decreased gradually in a PFK15 dose-dependent manner. And the cancer cells were halted in the G2/M phase, which was consistent with previous studies [24, 25]. Moreover, the similar cell cycle halt was also determined using FaDu cell line (Additional file 1: Figure S2c and d). A pervious study demonstrated the higher expression of PFKFB3 in cancer stem cells (CSCs) rather than the induced pluripotent stem cells (iPS), which might be able to be used to distinguish these two kind of stem cells [23]. Thus, tumor sphere formation assays were carried out to explore whether the stemness of HNSCC cells was changed after PFK15 treatment. Our results demonstrated that the number and the size of the tumor spheres formed by Cal27 single cells were dramatically decreased in the presence of PFK15 (Fig. 3f). And the results were also determined using FaDu cells (Additional file 1: Figure S2e). These results indicated the important role of PFKFB3 in the maintenance of cancer stem cells. We further explored whether PFK15 induced apoptosis in HNSCC cells. Annexin V-FITC/PI double staining was then performed to analyse the apoptosis of Cal27 cells after PFK15 treatment. The apoptotic cell population was quantitatively assessed *via* flow cytometric analysis (Fig. 3g). Although more apoptotic cells were detected in PFK15 treated group than in control group, PFK15 showed a weaker efficacy in inducing cell apoptosis than in suppressing cell proliferation. TUNEL Apo-Green detection assays were used to investigate apoptotic cell death by identifying fragmented DNA in Cal27 cells with the condensed green fluorescence in cell nuclei. As shown in Fig. 3h, the TUNEL positive staining of Cal27 cells increased after treatment with various PFK15 concentrations for 24 h. The expression levels of cell-proliferation- and apoptosis-related genes were examined by western blots (Fig. 3i). PFK15 significantly reduced the expressions of pRb, cyclin D1 and Bcl2, and upregulated the expression of cleaved caspase3 (CL-caspase3). In sum, targeting PFKFB3 by its selective suppressant PFK15 significantly suppressed cell proliferation and induced cell apoptosis in HNSCC.

### PFK15 inhibits cell migration and invasion of HNSCC cells

**Fig. 4:**
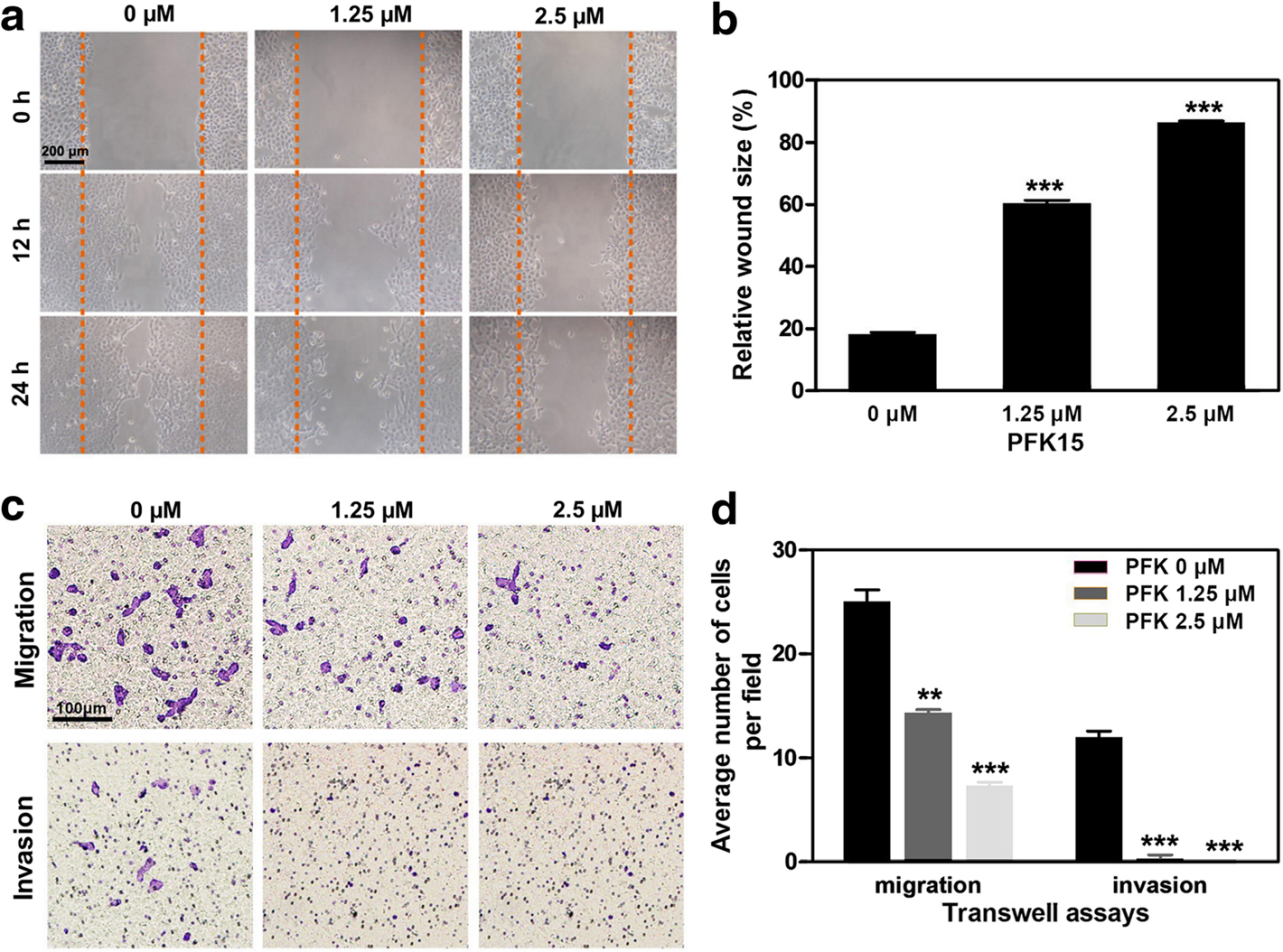
PFK15 reduces the migratory and invasive abilities of Cal27 cells. **a** The effects of PFK15 on the migration of Cal27 cells were tested by wound healing assays. **b** The quantitative data of the wound healing assays. **c** The migration and invasion of Cal27 cells that were treated with PFK15 were analyzed by transwell system. **d** The quantitative data of the migration and invasion assays using transwell chamber system. Mean ± S.E.M.; **P < 0.01, ***P < 0.001

To identify the potential roles of PKF15 in HNSCC local invasion and metastasis, the effects of PFK15 on cell migration and invasion were measured using wound healing assays and a transwell chamber system. As shown in Fig. 4a and b, PFK15 significantly reduced the migratory ability of Cal27 cells at 1.25 μM and 2.5 μM after 12 h treatment, at which concentrations PFK15 halted cell proliferation without causing significant cell death. The results from the transwell chamber system also showed that PFK15 suppressed the migration of Cal27 cells. We explored the invasive capability of Cal27 after PFK15 treatment by adding Matrigel on the upper chamber of the transwell system. As expected, PFK15 remarkably reduced the number of cells that crossed the Matrigel-coated semipermeable membrane (Fig. 4c and d). The quantitative data confirmed the abovementioned results. The suppressive effects of PFK15 on cell migration and invasion were also observed on FaDu cells (Additional file 1: Figure S3). In sum, using PFK15 to inhibit glycolysis in HNSCC cells could also dramatically suppress cell migration and invasion, suggesting that blockage of PFKFB3 was a promising prospect against tumor metastasis in HNSCC.

### PFK15 significantly impairs the invadopodia formation of HNSCC cells

**Fig. 5:**
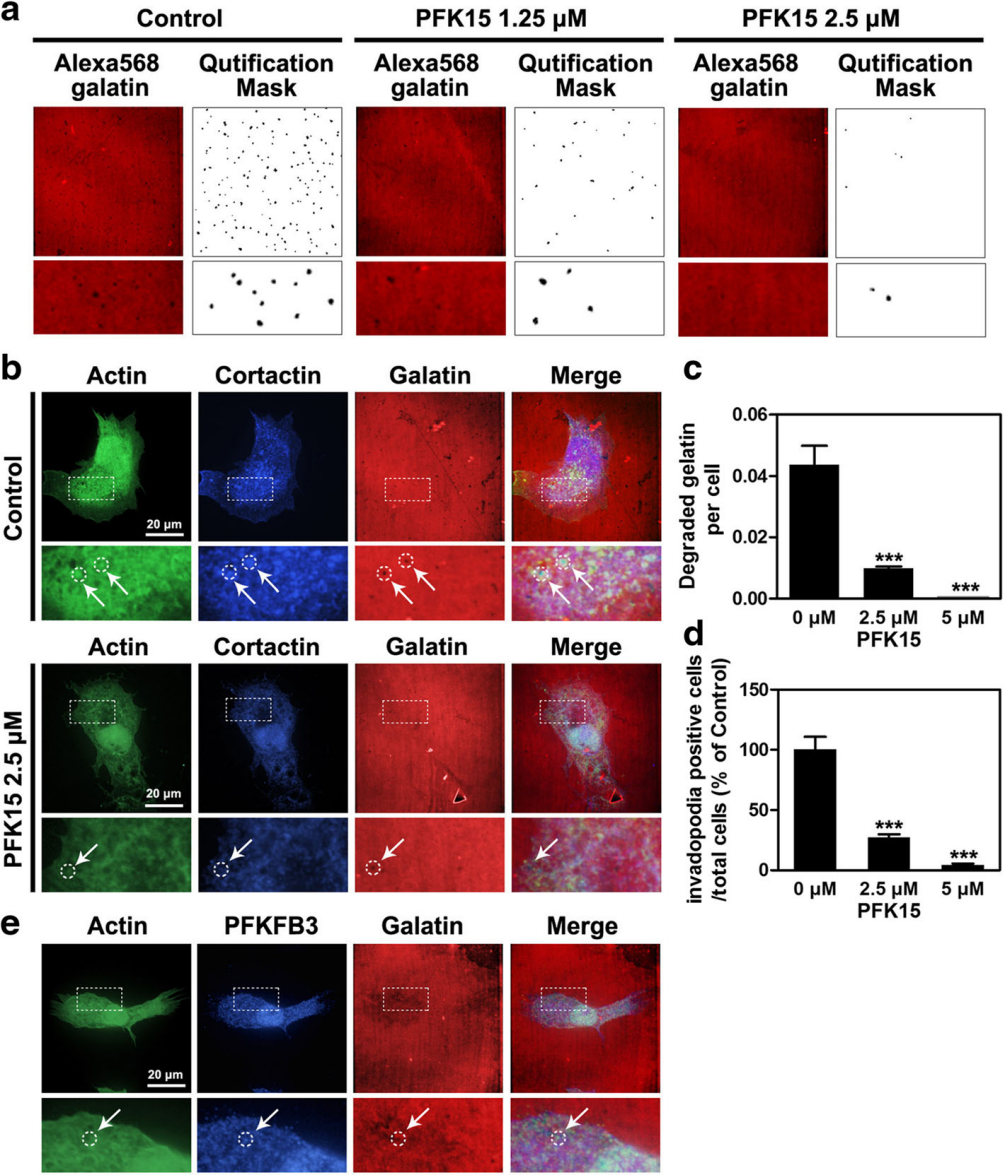
PFK15 impairs the formation and function of invadopodia in Cal27 cells. **a** The extracellular matrix (ECM) degradation ability of Cal27 cells after PFK15 treatment were measured by seeding tumor cells on the Alexa568-gelatin coated coverslips. **b** PFK15 impairs the formation of invadopodia inCal27 cells, which were detected by co-localisation of cortactin, F-actin and the black holes on the Alexa568-gelatin coated coverslips. **c** The quantitative data of the degraded matrix on the Alexa568-gelatin coated coverslips. **d** The quantitative data of the invadopodia formation per Cal27 cells. **e** The localisation of PFKFB3 in the invadopodia of Cal27 cells. Mean ± S.E.M.; ***P < 0.001

After proving that the blockage of glycolysis by PFK15 could suppress cell migration and invasion in HNSCC, we investigated whether targeting PFKFB3 impaired the function of invadopodia in HNSCC cells. The invadopodia formation of Cal27 cells was analysed by plating cells on the coverslips coated with Alexa568-labeled gelatine matrix, and the black holes that reflected ECM degradation were observed under a confocal laser scanning microscope. After using the quantification mask by virtue of Image J software, we found that the number and area of dark spots in Alexa568-labelled gelatin were significantly reduced after PFK15 treatment, which suggested a significant reduction in the ECM degradation ability of Cal27 cells (Fig. 5a). Such reduced ECM degradation ability reflected the impaired invadopodia function. Through the immunofluorescent staining of F-actin and cortactin—the core components of invadopodia, we further analysed the assembly of this F-actin-rich membrane protrusion. As shown in Fig. 5b, the co-location of F-actin and cortactin in Cal27 cells as well as the black holes in the Alexa568-labeled gelatine matrix indicated the formation of the invadopodia in Cal27 cells with ECM degradation capability. The obviously decreased number of dots in PFK15-treated Cal27 cells suggested the remarkably suppressed formation of invadopodia. The quantitative analysis results indicated that PFK15 reduced the ECM degradation ability of Cal27 cells by more than half at 2.5 μM and almost completely suppressed the ECM degradation of Cal27 cells at 5 μM (Fig. 5c). The quantitative data of invadopodia positive/total cells confirmed that PFK15 treatment significantly decreased the invadopodia of Cal27 cells (Fig. 5d).

A recent study demonstrated that PFKFB3 could regulate the protrusions of endothelial cells by binding to actin and generating ATPs for assembling protrusions. Therefore, we tested the subcellular localisation of PFKFB3 in Cal27 cells. The fluorescent images clearly showed that although most of PFKFB3 was localised in the nuclei of the tumor cells, the positive staining of PFKFB3 could also be detected in the invadopodia (Fig. 5e). Our supplementary data suggested that PFK15 treatment also impaired the lamellipodia of Cal27 cells (Additional file 1: Figure S4a and b). The positive staining of PFKFB3 was also detected in the lamellipodia clearly. By contrast, the visualisation of mitochondria using COX IV revealed that all the mitochondria were localised around the nuclei of Cal27 cells and excluded from lamellipodia (Additional file 1: Figure S4c). The ATPs generated through oxidative phosphorylation (OXPHOS) might not infiltrate into the formation of these protrusions. In sum, PFK15 evidently impairs the migration and invasion of tumor cells by decreasing the functional invadopodia and lamellipodia formation.

### PFK15 inhibits tumor growth in a HNSCC xenograft mouse model

**Fig. 6:**
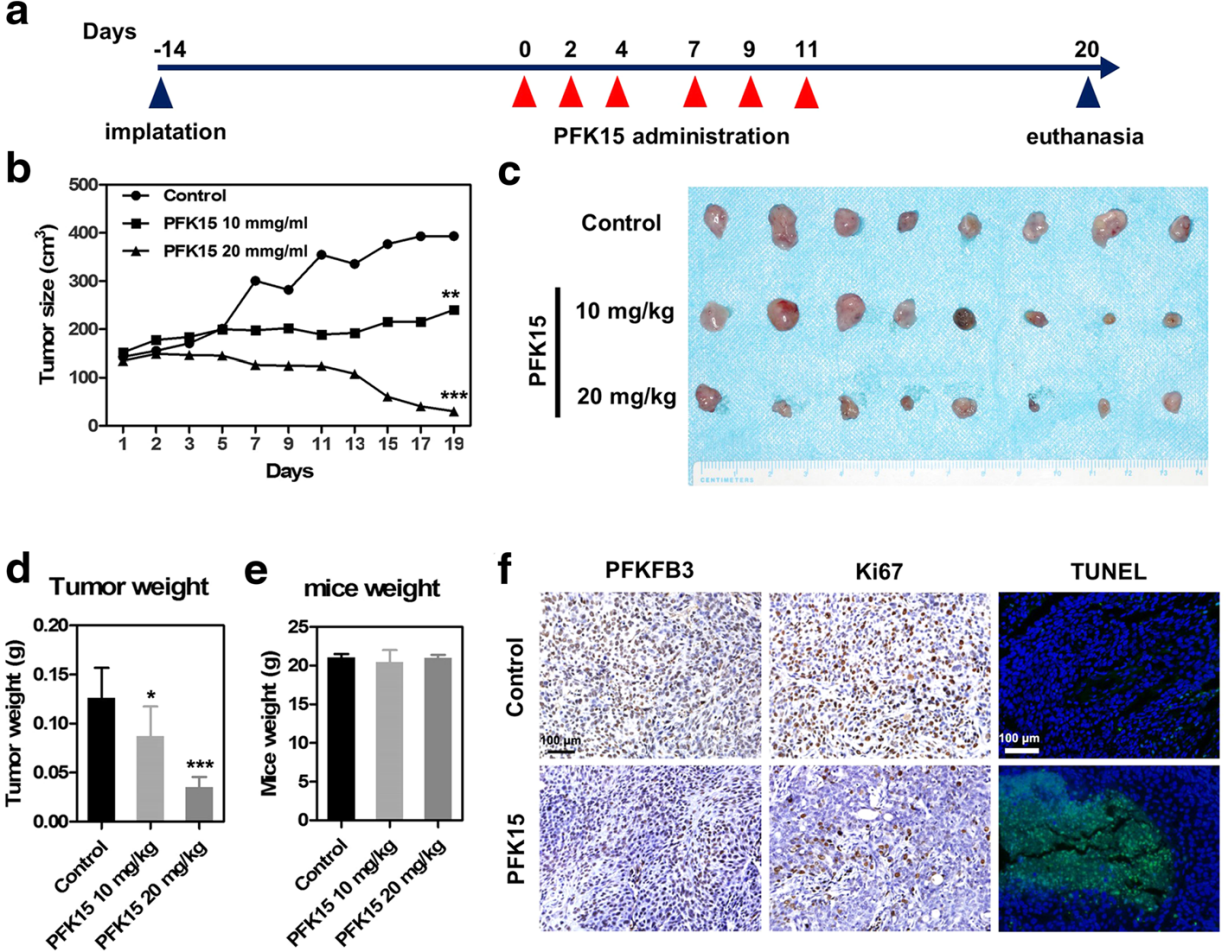
PFK15 inhibits the tumor growth in a HNSCC xenograft mice model. **a** The nude mice bearing xenograft were treated with 10 mg/kg and 20 mg/kg PFK15 intraperitoneally (i.p) every other day for 2 weeks. **b** Tumor growth was measured every other day. **c** Photos of the xenograft harvested from the nude mice after treatment with PFK15. **d** The tumor weights were measured after harvest from the carcinoma-bearing mice. **e** The mice weights were measured to examine the systematic toxicity of PFK15. **f** The expression of PFKFB3 and Ki67 were detected by immunohistochemistry, and the apoptotic cell death were examined by TUNEL assays. Mean ± S.E.M.; *P < 0.05, **P < 0.01, ***P < 0.001

To verify the therapeutic potentials of PFK15 in HNSCC treatment, a HNSCC xenograft model was established by subcutaneously injecting Cal27 cells into the flanks of the nude mice according to our previous study [20]. After 14 days of the injection of tumor cells, the mice received treatment, and the tumor volume and mice weights were measured every other day. Figure 6a showed the tumor cells implantation and drug administration strategies. The tumor growth curve revealed that PFK15 significantly slowed the development of the tumors at a 10 mg/kg concentration, but gradually reduced the tumor volume and almost completely abolished the tumor development at a 20 mg/kg concentration (Fig. 6b). After a 3-week PFK15 treatment, the mice were euthanized and the tumors were harvested. Figure 6c showed the tumors excised from each treatment group. The tumor weights shown in Fig. 6d further confirmed the excellent antitumor effects of PFK15, especially at a 20 mg/kg concentration. The body weights of mice after PFK15 treatment were comparable to those of the mice treated with PBS (control group), thereby suggesting the limited systemic toxicity of PFK15 during the treatment (Fig. 6e). The specimens from the xenografts were stained with PFKFB3, Ki67 and TUNEL. The immunohistochemistry results revealed that PFK15 did not affect the expression level of PFKFB3, but remarkably reduced the expression of Ki67 whilst inducing cell apoptosis in vivo (Fig. 6f). Therefore, targeting PFKFB3 by virtue of its selective suppressant PFK15 significantly impeded the development of HNSCC and even abolished the progression of HNSCC in a xenograft mouse model.

### PFK15 reduces the metastatic potential of HNSCC cells in vivo

**Fig. 7:**
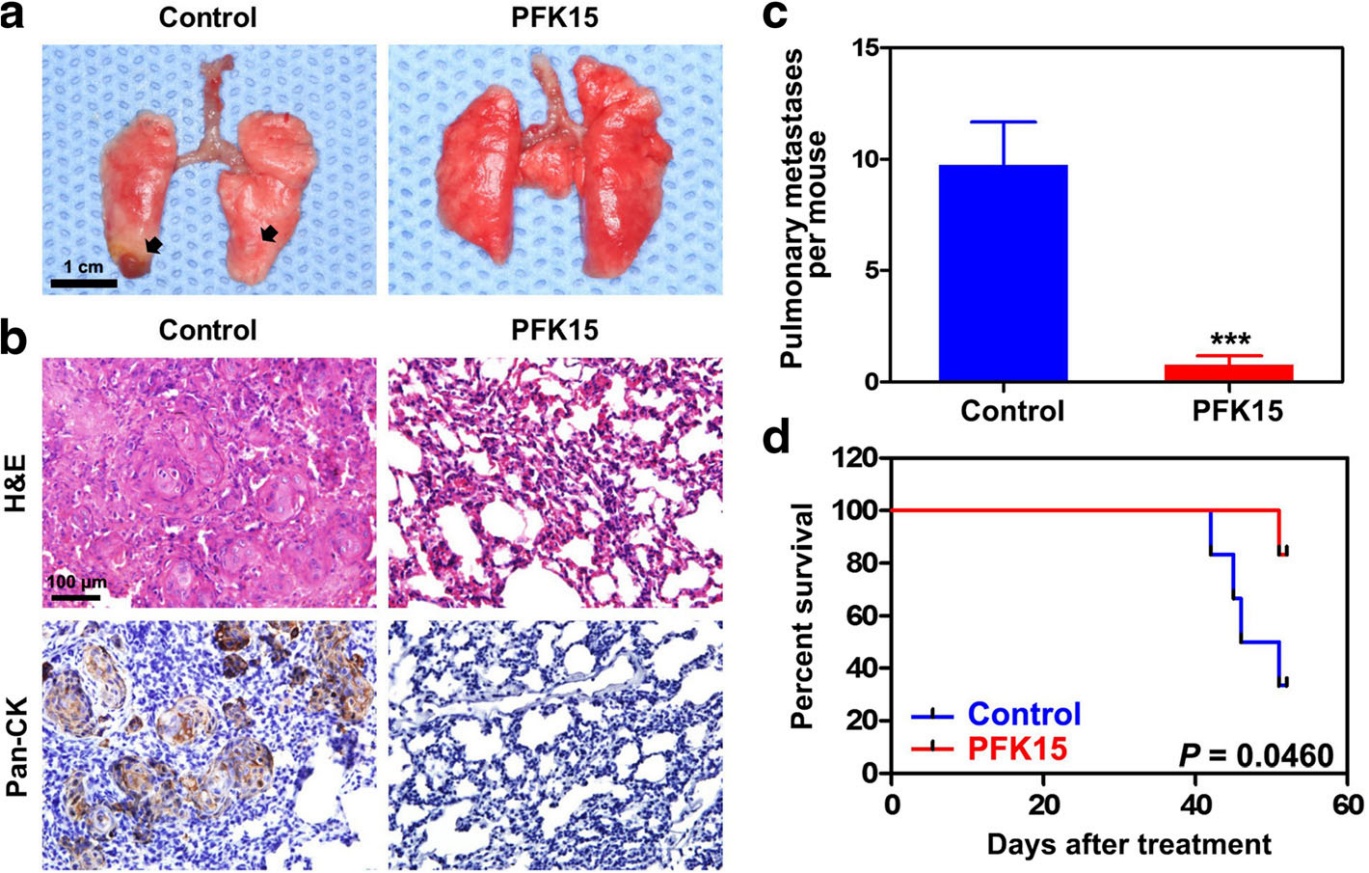
PFK15 prevents HNSCC distant metastasis in a HNSCC metastasis nude mice model. **a** Representative photos of the lung harvested from the mice bearing HNSCC metastasis treated with or without PFK15. **b** The metastatic nodules in the lung were determined by hematoxylin eosin (H & E) staining and human pan-CK immunohistochemical staining. **c** The quantitative of data of lung metastasis by counting the micrometastatic nodules per mice. **d** Kaplan-Meier survival curve of the mice treated with or without PFK15 were depicted. Mean ± S.E.M.; ***P < 0.001

To determine the potential therapeutic effects of PFK15 in tumor metastasis, a HNSCC metastatic mice model was established by injecting Cal27 cells into female BALB/c nude mice *via* tail vein. Two weeks after injection of tumor cells, 10 mg/kg PKF15 was administrated *via* intraperitoneal injection (every other day, 3 days/week for 2 weeks). Fifty days after the first PFK administration, the mice were euthanized and their lungs were harvested. The representative photos of the lungs harvested from the mice suggested that the metastasis nodules were dramatically decreased in mice with PFK15 treatment compared with those of the mice in the control group (Fig. 7a). Microscopic metastases to the lung were confirmed *via* H & E staining and pan-CK immunohistochemistical staining. As shown in Fig. 7b, the compact cell aggregations, which were further evidenced as tumor cells because of their positive staining of human pan-CK, were frequently observed in the lungs of the mice without any treatment, and the cell aggregations were much less and smaller in the lungs of the mice treated with PFK15. The lungs of each mouse from the control group had approximately 6 to 15 micrometastatic foci. By contrast, less than three micrometastatic foci were found in the lungs of three mice treated with PFK15, while the lungs of the other mice were free from metastasis. The incidence of metastasis was measured by the number of pulmonary metastatic clones (Fig. 7c), which confirmed the reduced metastatic ability of Cal27 cells in the model after PFK15 treatment. The survival curves demonstrated that PFK15 treatment extended the life expectancy of the mice suffering from the metastasis of Cal27 cells (Fig. 7d). In sum, the administration of PFK15 significantly prevents the distant metastases formation of HNSCC cells, thereby extending the life expectancy of these mice. This finding is consistent with the migration and invasion suppressive effects in the in vitro assays.

## Discussion

The metabolic switch from OXPHOS to aerobic glycolysis in cancer cells has been defined by Hanahan and Weinberg in 2011 as an emerging hallmark of cancer [26]. Despite its low ATP-generation efficacy, the increased glycolysis accompanied with a markedly increased uptake and utilisation of glucose in cancer cells could provide sufficient energy for cell survival and allowed for the diversion of glycolytic intermediates into various biosynthetic pathways, thereby facilitating the biosynthesis of the macromolecules and organelles required for assembling new cells. Many studies reveal that the execution of aggressive tumor agenda required a specific rewiring of the metabolic flux. The relationship between the glycolysis and metastasis in cancers has been increasingly acknowledged [27]. By targeting one of the most important glycolytic rate-limiting enzymes, PFKFB3, using its selective inhibitor PFK15, we proved that the blockage of glycolysis in HNSCC cells not only halted tumor growth by inhibiting cell proliferation but also alleviated cancer metastasis by suppressing the formation of protrusions. By establishing xenograft mice models and metastatic mice models, the aforementioned conclusions were further supported in vivo, validating the targeting of PFKFB3 as a promising strategy for HNSCC treatment.

Although the relationship between glycolysis and metastasis during cancer progression remains unknown, glycolysis is generally believed to be involved in several steps of metastasis. The extracellular acidosis (low pHe), which was associated with an elevated glycolytic flux due to the production and exportation of lactate through glycolysis, was considered a crucial factor for the detachment of tumor cells from neighbouring cells or ECM [28]. Extracellular acidosis also stimulated the secretion and/or activation of several hydrolases, including cathepsin and matrix metalloproteinases, which degraded the ECM components. The increased lactate in a microenvironment taken up by endothelial cells stimulated the secretion of interleukin (IL)-8 by activating the NF-κB pathway and promoting the formation of novel blood vessels that provide routes for cancer metastasis to distant organs. The accelerated glycolytic flux shunted the intermediates into the pentose phosphate pathway (PPP), which not only supported tumor cell proliferation but also facilitated tumor metastasis by preventing tumor cells from detachment-induced apoptosis (anoikis) during circulation [27, 29]. A recent study showed that PPP enhanced the tyrosine phosphorylation/activation of the HGF receptor, c-MET, which promoted cell migration and invasion by activating downstream pathways [30]. Glycolysis is also indispensable in the production of high amounts of ATP in these protrusions, which ensure a sufficient energy demand for dynamic cytoskeleton remodeling during cell migration and invasion. Another study revealed that the blockage of glycolysis, instead of mitochondrial OXPHOS, impaired the formation and function of invadopodia in several cancer cells [15]. Several glycolytic enzymes, including glyceraldehyde 3-phosphate dehydrogenase (G3P﻿DH), pyruvate kinase M2 (PKM2) and lLactate dehydrogenase A (LDH-A), were also found in the specialised protrusions, including lamellipodia and invadopodia [15].

Here, we observed that the blockage of glycolysis by targeting PFKFB3 could suppress the migration and invasion of HNSCC cells, which might be attributed to the reduced formation of invadopodia and lamellipodia. This study is the first to reveal the compartmentalisation of PFKFB3 in invading protrusions in cancer cells, as we found the co-localisation of PFKFB3 and the aggregation of F-actin (in both invadopodia and invadopodia) and ECM degradation spots (in invadopodia). This finding was consistent with that of a previous study, in which PFKFB3 was localised in the lamellipodia in endothelial cells [31]. The distribution of mitochondria only around the nuclei suggested that glycolysis might be main energy source for the assembly of invadopodia as proven in another study [15]. Importantly, we provided strong evidence that targeting PFKFB3 by PFK15 administration significantly reduced the lung metastases of the HNSCC cells in a mice model, and extended the life span of the mice. Therefore, targeting PFKFB3 offers a promising approach for preventing metastasis, and further investigations must be conducted to reveal precisely the molecular details during the formation and functionalization of invadopodia.

The persistence of aerobic glycolysis in many cancers provides a wide range of potential targets for therapy. We chose PFKFB3 as our target for several reasons. Firstly, PFKFB3 was widely expressed in several cancer types, and we here confirmed the high expression of PFKFB3 in HNSCC tissues but not in the adjacent normal tissues. Secondly, compared with the widely used glucose analogue 2-deoxy-D-glucose (2-DG), which may cause severe toxic and systemic adverse effects as its nearly inhibiting glycolytic activity completely [32], the systemic administration of 3PO, a small molecule antagonist of PFKFB3, only results in the partial and transient reduction of glycolysis without producing severe toxicity in normal tissues [12]. Developed by Clem et al. as a 3PO derivative in 2013 [16], PFK15 demonstrated approximately 100-fold more activities against PFKFB3 compared with 3PO. Thirdly, the blockage of PFKFB3 could inhibit the pathological angiogenesis without affecting the normal blood vessels [12]. Aberrant angiogenesis not only contributed to tumor growth by supporting nutrients and oxygen but was also involved in distant metastasis because of its high permeability. PFK15 significantly reduced the formation of novel blood vessels in the HNSCC xenografts (data not shown), which might contribute to the suppression of tumor growth and the alleviation of distant metastasis. Apart from mediating glycolysis acceleration, PFKFB3 might also involve in other tumor progressions. A previous study reported that PFKFB3 in the nucleus-generated F26BP could activate cyclin-dependent kinases and result in the phosphorylation and degradation of the Cip/Kip protein p27, thereby accelerating cell cycle and promoting cell proliferation [33]. And of interest, a recent study showed PFKFB3 controled human tongue tumor growth by responding to the circadian clock outputs [34].

## Conclusions

In sum, our study is the first to report that targeting PFKFB3 using its selective antagonist PFK15 not only halts the cell proliferation of HNSCC cells but also impairs their motilities for distant metastasis. The impaired motility of HNSCC cells might be attributed to the fact that the blockage of glycolysis in HNSCC impeded the formation of functional invadopodia and lamellipodia, which were the specialised protrusions that facilitate cell migration and invasion. By establishing HNSCC xenograft mice models and metastatic mice models, we further tested the therapeutic efficacy of PFK15 on HNSCC in vivo, and proved that the blockage of glycolysis by targeting PFKFB3 not only suppressed tumor growth but also alleviated the distant metastasis of HNSCC, reflecting the promising application of PFK15 for HNSCC treatment.

## Additional file

Additional file 1: Additional Materials and Methods. **Figure S1.** PFK15 inhibits the cell growth and glycolytic activity of FaDu cells. **Figure S2.** PFK15 suppresses cell proliferation and halts cell cycle in FaDu cells. **Figure S3.** PFK15 decreases the migratory and invasive abilities of FaDu cells. **Figure S4.** PFK15 impairs the formation lamellipodia in Cal27 cells. (DOC 910 kb)

## Abbreviations

ECM: Extracellular matrix
OXPHOS: Oxidative phosphorylation
PFK15: 1-(4-pyridinyl)-3-(2-quinolinyl)-2-propen-1-one
PFKFB3: Phosphofructokinase-2/fructose-2, 6-bisphosphatase 3
