# Supplementary material for: Blockage of glycolysis by targeting PFKFB3 suppresses tumor growth and metastasis in head and neck squamous cell carcinoma
Source: J Exp Clin Cancer Res. 2017 Jan 7;36:7. doi: 10.1186/s13046-016-0481-1 (PMC5219669; doi:10.1186/s13046-016-0481-1)
Supplement: Additional file 1: — Additional Materials and Methods. Figure S1. PFK15 inhibits the cell growth and glycolytic activity of FaDu cells. Figure S2. PFK15 suppresses cell proliferation and halts cell cycle in FaDu cells. Figure S3. PFK15 decreases the migratory and invasive abilities of FaDu cells. Figure S4. PFK15 impairs the formation lamellipodia in Cal27 cells. (DOC 910 kb) [file 13046_2016_481_MOESM1_ESM.doc]

**ADDITIONAL MATERIALS AND METHODS**

**F26BP assays**

Intracellular level of fructose 2, 6-bisphosphate (F26BP) was measured as previously reported. Briefly, cells were harvested and lyzed in 20 volumes of 0.05 N NaOH, and 1 volume of 0.1 N NaOH to obtain a pH > 11, followed by incubation at 80 °C for 5 min. Cell extracts were then neutralized to pH 7.2 with acetic acid in 20 mM Hepes. And the samples were incubated at 25 °C for 2 min in the mixture containing 50 mM Tirs, 2 mM Mg^2+^, 1 mM fructose 6-phosphate (F6P), 0.15 mM NAD, 10 U/L PPi-dependent PFKi, 0.45 KU/L glycerol-3-phoshate dehydrogenase. In total, 0.5 mM pyrophosphate was added and the rate of change in absorbance (OD = 339 nm) per min was followed for 5 min. F26BP was calculated based on a calibration curve produced by measuring 0.1 to 1 pmol of F26BP and normalized to total cellular protein. All the chemicals used were purchase from Sigma (St Louis, MO).

**Glucose uptake measurement assay**

The glucose uptake was detected using the Glucose Uptake Cell-Based Assay Kit according to the manufacturer’s instructions. 2-NBDG, a fluorescence-labeled deoxyglucose analog, was employed in this kit as a probe for detecting of the glucose uptake. The fluorescence intensity was recorded using a fluorescence microscopy (Olympus, Japan) and a microplate reader (Thermo, Waltham, MA, USA).

**L-Lactate detection assay**

To determine the generation of intracellular lactic acid, cells incubated with different concentrations of PFK15 were collected and assayed based on the manufacturer's instructions of the Cayman’s L-Lactate Assay Kit, then detected by a fluorometer (Thermo, Waltham, MA, USA).

**ATP detection assay**

Different cell groups were collected and the cellular ATP levels were measured using a firefly luciferase-based ATP Assay Kit according to the manufacturers' instructions. Luminescence in the ATP assay was measured using the GloMax^TM^20/20 Luminometer (Promega, Madison, USA) and recorded by Microsoft® Excel Software.

**MTT assay**

The cytotoxic effect of PFK15 on cell viability was measured by MTT assay. Briefly, Cal27 and FaDu cells were plated into 96-well plates at a density of 2×10^4^ per well for overnight incubation, and then exposed to PFK15 at various concentrations (0, 1.25, 2.5 and 5μM) for 12, 24, 48 and 72h. Then, 100μl DMEM containing 10μl MTT (5mg/ml) was added to each well and incubated for another 4h at 37°C. After the supernatant was carefully discarded, the remaining precipitate was mixed and dissolved with 200μl of DMSO per well by gently pipetting to prevent air bubbles. Then the absorbance was assessed at 490 nm using a 96-well microplate reader (BioTek, Winooski, VT, USA).

**Cell clone formation Assay**

The exponentially growing cells were collected and seeded in 6-well plates at a density of 2000 cells per well. Culture medium with different indicated PFK15 concentration was added to the well after overnight growth and changed at regular time intervals. Cal27 cells were incubated at 37°C for 14 days to allow colonies formation. Then the adherent cells were washed three times with PBS, fixed with 4% paraformaldehyde and stained with 5% crystal violet for 20 min. Surviving colonies (>50 cells per colony)were counted and the plates were instantly photographed. The experiments were repeated for three times in triplicate and the mean was calculated.

**Cell cycle analysis**

HNSCC cells were exposed to PFK15 of indicated concentrations as mentioned above for 24h. Then cells were fixed in cold 70% ethanol for 30 min at 4°C, and washed with PBS for twice. After centrifugation at 800 g for 5 min, the cells were resuspended and incubated with 100μg/ml RNase A and 50μg/ml propidium iodide (PI) for 30 min. And the DNA content was performed by the FACScan Calibur FlowCytometerusing at least 20,000 cells per sample and data were analyzed using the FlowJo software.

**Tumor sphere formation assays**

For tumor spheres formation assays, Cal27 and FaDu cells were seeded at 2×10^3^ cells/well in 6-well ultralow adherence plates (Corning, Tewksbury, MA) in DMEM/F12 containing 20ng/ml recombinant epidermal growth factor (EGF), 20ng/ml basic fibroblast growth factor (bFGF), 2% B27 supplement (Invitrogen, Carlsbad, CA). Then PFK15 were added into the culture system at the indicated concentrations. Suspension cultures were incubated for 7 days, and the formed colonies were captured.

**Determination of apoptosis**

Induction of apoptosis was determined as followed: (a) induction of apoptosis by PFK15 was morphologically assessed by analysis of damaged nucleus with TUNEL Apo-Green detection kit; (b) the apoptotic cells were stained using the Annexin V-FITC apoptosis detection kit and quantitatively analyzed with FACScan Calibur FlowCytometer (Becton-Dickinson Biosciences, San Diego, CA, USA). At least 10,000 events per sample were acquired and analyzed at the rate of 50 to 500 events per second and data was analyzed by BD Cell Quest and WinMDI 2.9 software (Becton-Dickinson Biosciences, San Diego, CA).

**Wound healing assay**

Cal27 cells were seeded into 6-well culture plates at a density of 5×10^5^ cells per well. When the cells reached to 90% confluence, a scratch wound was made in the center of the plate with a sterile 200μl pipette tip. The cells were washed with PBS for 3 times to remove the floating cells, andthen incubated with fresh serum-free medium containing PFK15 at different concentrations (0, 1.25, 2.5 and 5μM) for 24 h. Photos were taken after 12h and 24h. The average migration distancewas analyzed and calculated using ImageJ1.42.

**Transwell migration and invasion assays**

Cell migration assays were performed using Transwell Boyden chamber system containing a polycarbonate filter (6.5mm diameter; pore size of 8μm, #3422). For invasion assays, 40µl matrigel was coated on the upper surface of chambers. Cal27 and FaDu cells were firstly cultured to grow to subconfluency (75-80%) and then serum-starved for 24h. Cell suspensions (6×10^5^ cells/ml) in 100μl serum-free medium were seeded into the upper chamber, while 800μl of DMEM with 10%FBS were added to the bottom chambers as chemoattractant. After incubation for 24h (36h for invasion assay), the cells that did not penetrate through the filters were carefully cleared with cotton swabs, followed by a gentle washing step. The migrated cells on the lower surface of the filter were fixed with 4% paraformaldehyde and stained with crystal violetfor visualization. Images of five randomly chosen fields at 200×magnification were captured from each membrane.

**ADDITIONAL FIGURES**

**Figure S1**


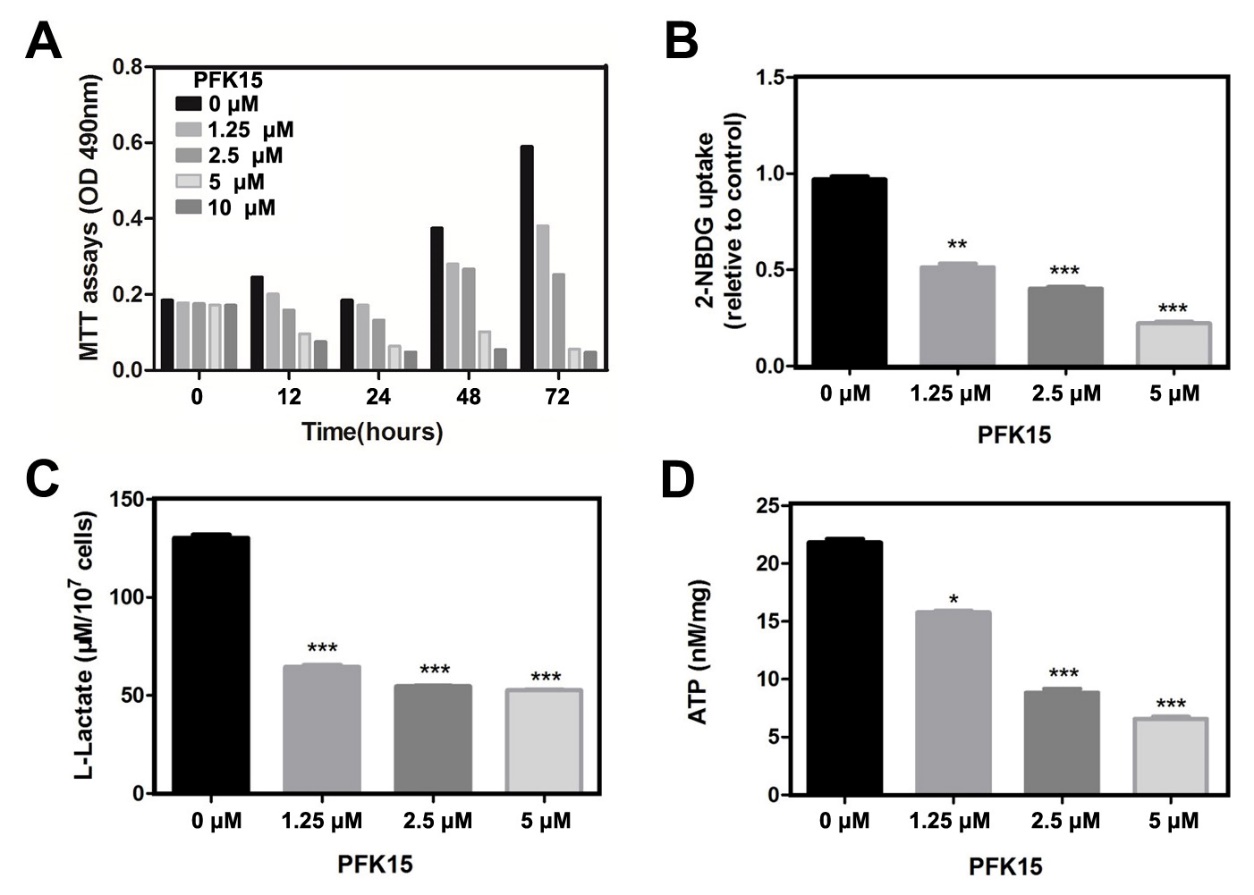


**Figure S1. PFK15 inhibits the cell growth and glycolytic activity of FaDu cells. a** PFK15 suppressed the cell viability of FaDu cells in a time- and concentration- dependent manner.**b** Glucose uptake was analyzed in FaDu cells using 2-NBDG.**c** Lactate production in FaDu cells treated with PFK15 were detected. **d** ATP generation in FaDu cells treated with PFK15 were measured.

**Figure S2**


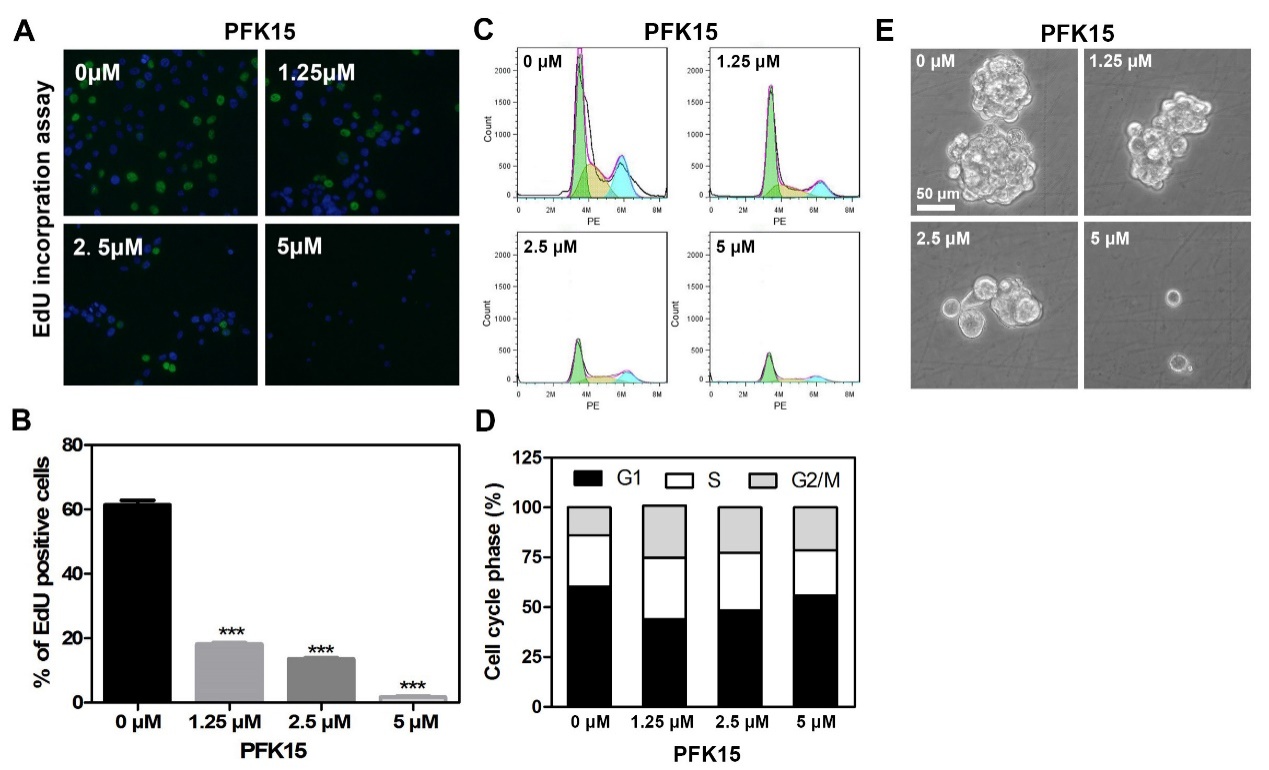


**Figure S2. PFK15 suppresses cell proliferation and halts cell cycle in FaDu cells. a** EdU incorporation assays indicated PFK15 suppressed cell proliferation. **b** The quantitative data of EdU incorporation assays in FaDu cells. **c** PI staining revealed that PFK15 halted cell cycle progression and induced G2 phase arrest. **d** The quantitative data of cell cycle analysis based on Dean-Jett-Fox model. **e** Tumor sphere formation assays indicated PFK15 destroyed the cancer stem cell population of FaDu cells. Mean ± S.E.M.; ****P*< 0.001.

**Figure S3**


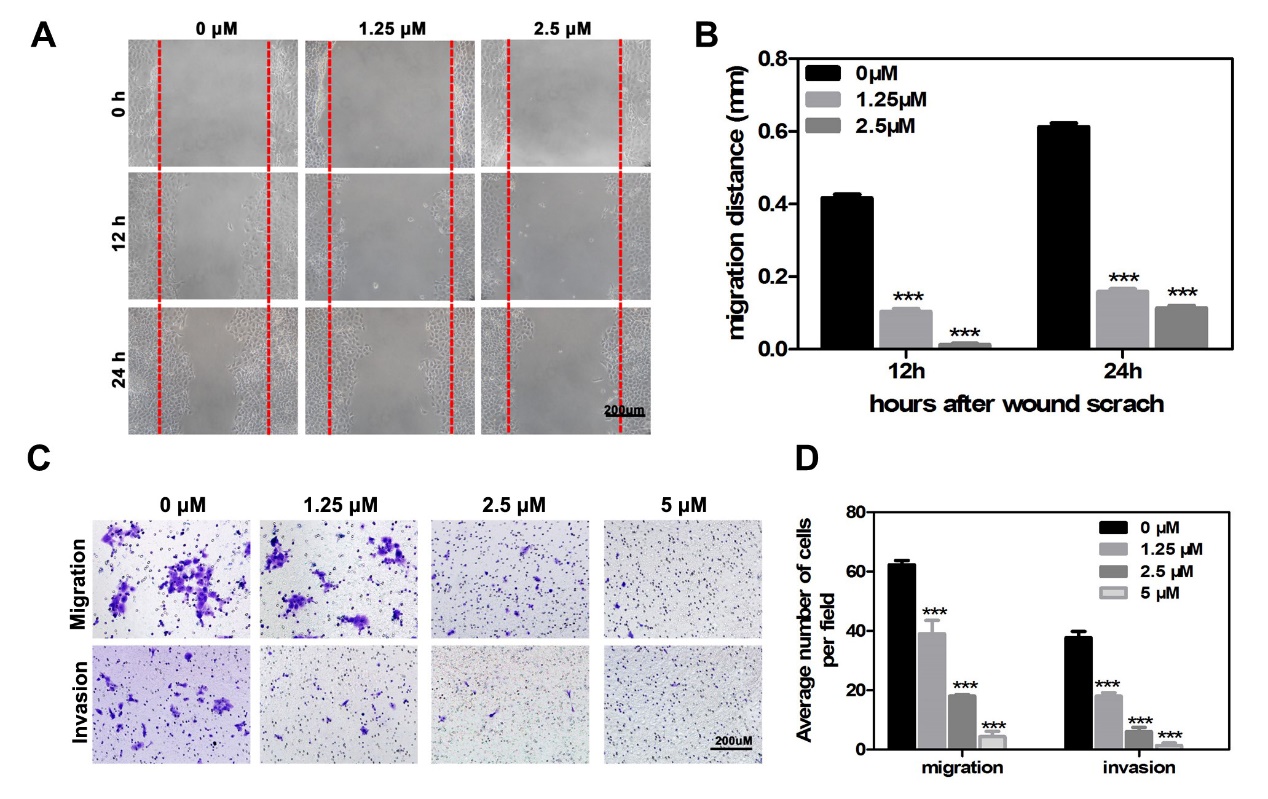


**Figure S3. PFK15 decreases the migratory and invasive abilities of FaDu cells. a** The effects of PFK15 on the migration of FaDu cells were tested by wound healing assays. **b** The quantitative data of the wound healing assays. **c** The migration and invasion of FaDu cells that were treated with PFK15 were analyzed by transwell system. **d** The quantitative data of the migration and invasion assays using transwell chamber system. Mean ± S.E.M.; ****P*< 0.001.

**Figure S4**


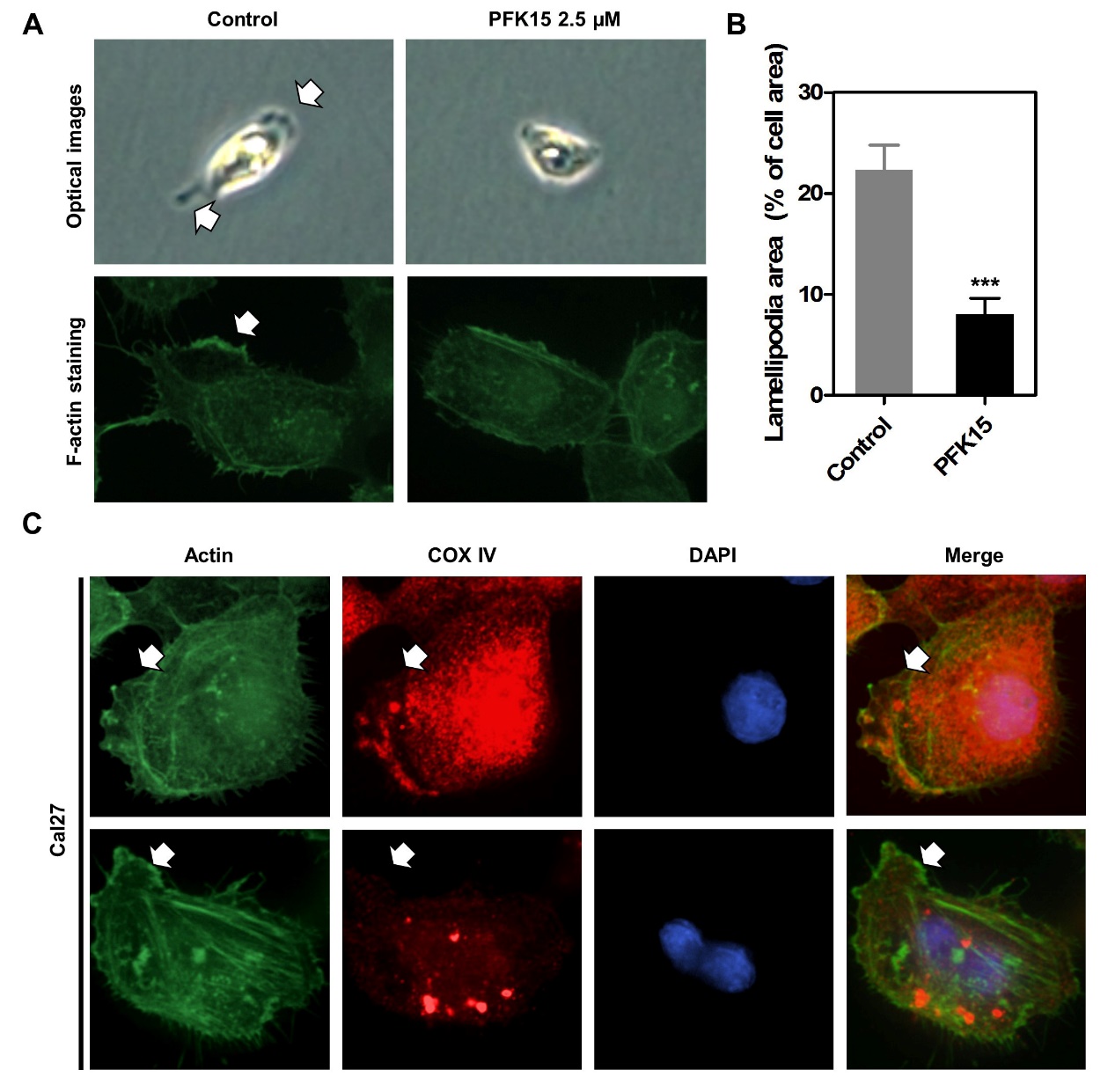


**Figure S4. PFK15 impairs the formation lamellipodia in Cal27 cells. a** The lamellipodia of Cal27 cells treated with or without PFK15 were detected under optical microscope and fluorescence microscope after FITC-phalloidine staining. **b** The quantitative analysis of lamellipodia in Cal27 cells. **c** The co-localization of PFKFB3 but not COX IV (the marker of mitochondria) in the lamellipodia were determined by three-colour immunofluorescence staining.
